# Positioning of Tacrolimus for the Treatment of Diabetic Nephropathy Based on Computational Network Analysis

**DOI:** 10.1371/journal.pone.0169518

**Published:** 2017-01-06

**Authors:** Constantin Aschauer, Paul Perco, Andreas Heinzel, Judith Sunzenauer, Rainer Oberbauer

**Affiliations:** 1 Department of Nephrology, Medical University of Vienna, Vienna, Austria; 2 Emergentec Biodevelopment GmbH, Vienna, Austria; 3 Department of Internal Medicine IV, Medical University of Innsbruck, Innsbruck, Austria; Istituto Di Ricerche Farmacologiche Mario Negri, ITALY

## Abstract

**Objective:**

To evaluate tacrolimus as therapeutic option for diabetic nephropathy (DN) based on molecular profile and network-based molecular model comparisons.

**Materials and Methods:**

We generated molecular models representing pathophysiological mechanisms of DN and tacrolimus mechanism of action (MoA) based on literature derived data and transcriptomics datasets. Shared enriched molecular pathways were identified based on both model datasets. A newly generated transcriptomics dataset studying the effect of tacrolimus on mesangial cells in vitro was added to identify mechanisms in DN pathophysiology. We searched for features in interference between the DN molecular model and the tacrolimus MoA molecular model already holding annotation evidence as diagnostic or prognostic biomarker in the context of DN.

**Results:**

Thirty nine molecular features were shared between the DN molecular model, holding 252 molecular features and the tacrolimus MoA molecular model, holding 209 molecular features, with six additional molecular features affected by tacrolimus in mesangial cells. Significantly affected molecular pathways by both molecular model sets included cytokine-cytokine receptor interactions, adherens junctions, TGF-beta signaling, MAPK signaling, and calcium signaling. Molecular features involved in inflammation and immune response contributing to DN progression were significantly downregulated by tacrolimus (e.g. the tumor necrosis factor alpha (TNF), interleukin 4, or interleukin 10). On the other hand, pro-fibrotic stimuli being detrimental to renal function were induced by tacrolimus like the transforming growth factor beta 1 (TGFB1), endothelin 1 (EDN1), or type IV collagen alpha 1 (COL4A1).

**Conclusion:**

Patients with DN and elevated TNF levels might benefit from tacrolimus treatment regarding maintaining GFR and reducing inflammation. TGFB1 and EDN1 are proposed as monitoring markers to assess degree of renal damage. Next to this stratification approach, the use of drug combinations consisting of tacrolimus in addition to ACE inhibitors, angiotensin receptor blockers, TGFB1- or EDN1-receptor antagonists might warrant further studies.

## Introduction

Tacrolimus is a powerful immunosuppressive drug belonging to the group of calcineurin inhibitors (CNIs), which was introduced in clinical use to tackle organ rejection in solid organ transplantation [1]. Tacrolimus nephrotoxicity is a dose-dependent side effect and people have started to investigate the role of pharmacogenetics in tacrolimus pharmacodynamics by looking at single nucleotide polymorphisms in genes of the cytochrome P450 family as well as in ABC transporters in order to optimize dosing on an individual patient level [2].

Tacrolimus is used for the treatment of lupus nephritis and also a clinical trial in the context of IgA glomerulonephritis has recently been conducted [3][NCT01224028]. There is also evidence on the positive effects of tacrolimus on diabetic nephropathy available, both on the level of animal models but also in human subjects with one human trial reporting beneficial effects of tacrolimus in a combination therapy with valsartan on renal function in a set of patients with diabetic nephropathies [4][5][6]. These animal studies show that next to the anti-inflammatory effect of tacrolimus, there is evidence of direct effects on signalling cascades in renal cells. The effect of tacrolimus on individual molecules, especially components of the TGF-beta signalling cascade, in mesangial cells has been studied previously with negative as well as positive effects being reported [7][8][9]. Mesangial cells are a good in-vitro model in order to study mesangial proliferation, matrix accumulation, fibrosis, and glomerulosclerosis, all hallmarks of diabetic nephropathy [10].

In this study we investigated the effects of tacrolimus on pathophysiological mechanisms of diabetic nephropathy on the level of in-silico constructed mechanism of action and disease pathophysiology molecular models respectively. We furthermore searched for molecular markers showing the potential for identifying the cohort of diabetic nephropathy patients benefitting the most from the immunosuppressive properties of tacrolimus.

## Materials and Methods

### Molecular models of tacrolimus mechanism of action (MOA) and diabetic nephropathies pathophysiology

Molecular features affected by tacrolimus were extracted from scientific literature taking into consideration genes linked via NCBI gene2pubmed associations to publications which were annotated with the MeSH term “tacrolimus”. In addition deregulated transcripts were extracted from two published transcriptomics studies from Kern and colleagues as well as Maluf and colleagues focusing on the effect of tacrolimus on renal tissue, in particular on renal fibroblasts and post-kidney transplants, respectively [11][12]. Transcripts from scientific literature as well as from the two omics studies were further mapped to their Ensembl Gene IDs and the set of unique protein-coding Ensembl Gene IDs was used as input set for generating a tacrolimus mechanism of action molecular model. In brief these molecular features were mapped on a hybrid interaction network including protein-protein interaction data from IntAct, BioGrid, and Reactome together with computationally inferred relations [13]. The induced subgraph was extracted after mapping the features onto the network, including all molecular features holding an interaction to at least one other feature from the input set. The induced subgraph was further segmented into clusters of proteins using the Molecular Complex Detection algorithm following a procedure as described previously [14][15].

Construction of a diabetic nephropathy molecular model followed the same procedure as construction of the tacrolimus MoA model using as input features associated via gene2pubmed to publications holding “diabetic nephropathies” as major MeSH term.

A molecular pathway enrichment analysis was conducted for the two constructed molecular models using the Database for Annotation, Visualization and Integrated Discovery (DAVID) version 6.7 [16]. Results were corrected for multiple testing setting the false discovery rate to <5% for identification of significantly enriched molecular pathways. Disease specific molecular pathways like e.g. pathways in cancer or type II diabetes mellitus were excluded from the output thus focusing on molecular signalling cascades as well as metabolic pathways.

### Cell culture experiments

Primary human mesangial cells (Lonza, Clonetics, CC-2559) were cultured in MCDB-131 media (Gibco-invitrogen), supplemented with 10% FBS, 100U penicillin/ml 100ug/ml streptomycin and 2mmol L-Glutamine (all supplied from Gibco-Invitrogen) at 37°C in humid atmosphere with 5%CO_2_ in air. Cells were seeded onto six-well cell culture plates (Greiner Bio one) and were used for experiments at the fifth passage. Confluent cells were cultured in serum free media and FK 506 (Abcam) dissolved in DMSO (Sigma) in a final concentration of 1 μM for 24h. The control group was treated in serum free media and DMSO only and a total of four independent experiments were conducted. Trizol® and chloroform reagent was used to extract RNA (Invitrogen). The RNA pellet was dissolved in 10 μl sterile water and stored at –80°C.

### Transcriptomics profiling

Total RNA from human mesangial cells was reverse transcribed, labelled and hybridised to Affymetrix Human Gene (HuGene) 2.0 ST Array (Affymetrix) according to the manufacturer’s protocol. The oligo R package was used for pre-processing and normalization of expression data. The statistical analysis of microarrays (SAM) method available in the TIGR MultiExperimentViewer was used in order to identify differentially regulated transcripts between tacrolimus treated and untreated cells [17][18]. Transcripts were mapped to the respective Ensembl Gene IDs and the set of unique protein-coding genes was used for further analysis.The microarray data have been deposited in NCBI's Gene Expression Omnibus and are accessible through GEO Series accession number GSE84908 (https://www.ncbi.nlm.nih.gov/geo/query/acc.cgi?acc=GSE84908).

### Model interference analysis and biomarker annotation

The two constructed molecular models, namely the one representing tacrolimus MoA and the one reflecting diabetic nephropathy pathophysiology on a molecular network level were compared to each other with respect to molecular feature overlap as previously described [19]. Deregulated transcripts by tacrolimus in the in-vitro mesangial cell model were further mapped onto the diabetic nephropathy molecular model and considered as relevant when analyzing the effect of tacrolimus on diabetic nephropathy and also when further selecting biomarker candidates.

Biomarker evidence for diabetic nephropathy diagnosis and prognosis was gathered from scientific literature for molecular features in the diabetic nephropathies molecular model being affected by tacrolimus. Therefore molecular features were extracted from publications annotated with the major MeSH term “diabetic nephropathies” as well as the MeSH terms “biological markers” OR “genetic markers” neglecting profiling studies by excluding publications annotated with one of the following MeSH terms: “gene expression studies”, “microarray analysis”, “proteomics”, “metabolomics”, or “genome-wide association study”. Publications however had to be either of publication type “clinical trial” or annotated with “humans” or “disease models, animal”. Prognostic markers were extracted from publications further holding the MeSH term “prognosis”, whereas diagnostic markers were extracted from publications annotated either with “diagnosis”, “diagnosis, differential”, “early diagnosis”, or “diabetic nephropathies/diagnosis”. Next to considering molecular features assigned via gene2pubmed to the respective marker papers, we mined for molecular features using their respective gene names and considered perfect matches as hits.

## Results and Discussion

### Molecular models of diabetic nephropathy and tacrolimus MOA

583 molecular features could be extracted from scientific literature being associated with diabetic nephropathy. The constructed molecular model for diabetic nephropathies in total held 252 molecular features in 15 process units ranging in size from 3 to 58 molecular features. The top enriched molecular pathway of the 17 identified molecular pathways was *calcium signaling* with 28 molecular features out of the 252 being assigned to this pathway (p-value < 0.001). A set of signaling cascades were among the list of enriched molecular pathways like e.g. *Adipocytokine signaling* (p-value < 0.001), *MAPK signaling* (p-value < 0.001), *TGF-beta signaling* (p-value < 0.001), or *PPAR signaling* (p-value = 0.002). Next to these signaling cascades, molecular pathways linked to inflammation and immune response were enriched like for example *cytokine-cytokine receptor interactions* (p-value = 0.002), *Toll-like receptor signaling* (p-value 0.002), or *chemokine signaling* (p-value = 0.003). In addition the *renin-angiotensin system* (p-value < 0.001) as well as the *complement and coagulation system* (p-value < 0.001) were enriched with molecular features from the constructed DN molecular model.

**Fig 1:**
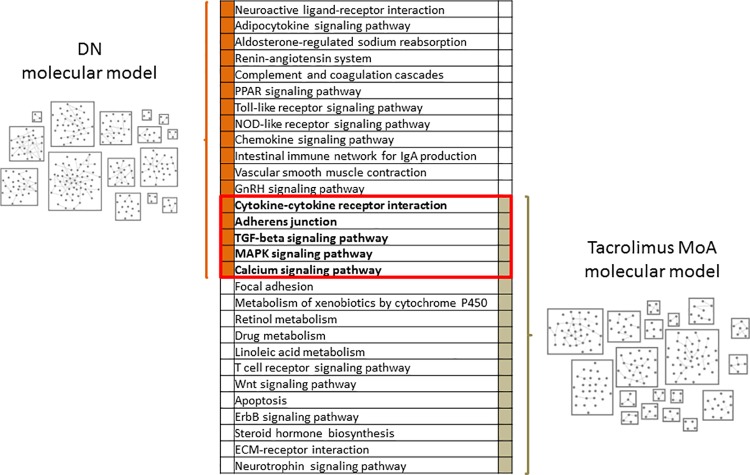
Molecular models. Schematic representation of the constructed DN molecular model and the tacrolimus MoA molecular model with the list of significantly enriched molecular pathways based on the two molecular model features sets. Molecular pathways in bold font within the red border were significantly enriched in molecular features of both molecular model sets. Each box in the molecular model representations depicts one process unit, characterized by highly inter-connected proteins, with individual nodes representing protein coding genes.

The tacrolimus MoA molecular model held 209 molecular features assigned to 19 process units ranging in size from 3 to 38 molecular features. From the 785 unique protein coding genes forming the input for model construction, 582 protein-coding genes originated from the two transcriptomics studies with 217 genes coming from literature mining for tacrolimus-associated molecular features. Five of the enriched molecular pathways in DN were also significantly enriched based on the set of molecular features in the tacrolimus MoA molecular model, namely cytokine-cytokine receptor interactions, adherens junctions, TGF-beta signaling, MAPK signaling, as well as calcium signaling (Fig 1).

The use of heterogeneous data sets for constructing the tacrolimus MoA molecular model could be seen very critically but can also be seen from a positive angle. With input data coming from fibroblasts as well as from post-transplant kidneys it is not possible to reconstruct cell-specific molecular pathways, but on the other hand it allows assessing the effect of tacrolimus on kidney as a whole. Following this line we furthermore assessed the effect of tacrolimus on mesangial cells using gene expression profiling.

### Tacrolimus effect on mesangial cells

21534 transcripts remained after preprocessing and normalization steps which were used for hierarchical cluster analysis in order to see whether there was an effect of tacrolimus on mesangial cells in culture. 1—Pearson correlation and average linkage were used as distance measure and linkage rule. In the cluster dendrogram, a clear separation between the tacrolimus-treated and the untreated control samples was detectable although absolute differences in gene expression between samples were only minor. Nevertheless 222 differentially regulated transcripts were identified using the SAM with a false discovery rate of lower than 5%. 154 transcripts were upregulated after tacrolimus treatment with 68 being downregulated. The deregulated transcripts could be mapped to 204 unique protein coding genes on the level of Ensembl Gene IDs with 141 upregulated and 63 downregulated genes.

### Interference and biomarker analysis

**Fig 2:**
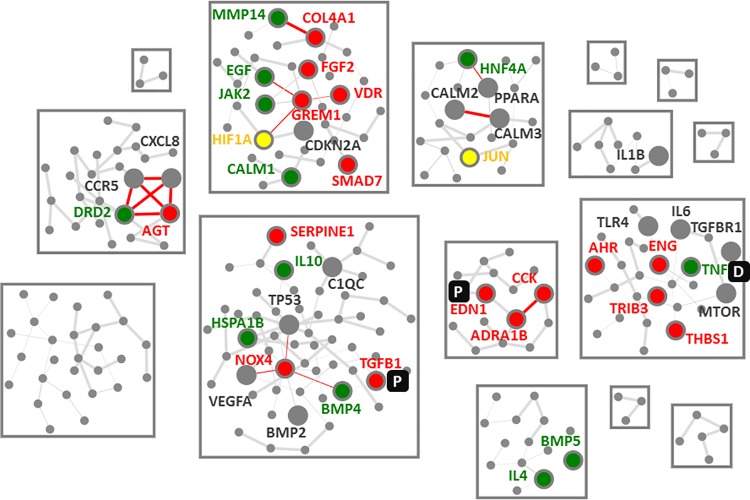
Interference network. Depicted is the diabetic nephropathy molecular model with molecular features affected by tacrolimus being displayed with larger nodes and annotated with the official Gene Symbols. Molecular features upregulated by tacrolimus are highlighted in red, with downregulated molecular features displayed in green. Molecular features with contradictory evidence, i.e. up- and down-regulated are depicted in yellow. Annotated grey nodes depict molecular features being associated with effects of tacrolimus, in most cases SNPs in these genes having impact on tacrolimus efficacy. Prognostic and diagnostic biomarkers are indicated by a P or D respectively.

39 of the 252 individual molecular features of the DN molecular model were also part of the tacrolimus MoA molecular model thus being affected by tacrolimus. In addition six further molecular features of the DN molecular model could be identified as deregulated after tacrolimus treatment in mesangial cells (Fig 2).

Three molecular features had annotation evidence as marker for diabetic nephropathy, with TNF being reported as diagnostic marker for DN and TGFB1 and EDN1 showing evidence as prognostic marker for DN.

A set of molecular features involved in inflammation and immune response like the tumor necrosis factor alpha (TNF), interleukins 4 and 10 (IL4, IL10), or the heat shock protein family A (Hsp70) member 1B (HSPA1B) were all downregulated by tacrolimus.

Inflammation is recognized as a major factor in diabetic nephropathy [20][21], subsequently leading to abnormal tissue repair and progressive fibrosis with alteration of tissue structure and loss of kidney function [22]. Recent studies indicate that treatment with anti-inflammatory agents leads to reduction of subclinical inflammation, giving account for the potential positive effect of tacrolimus on the course of diabetic nephropathy [23][24][25].

TNF was previously reported as biomarker for diagnosing diabetic nephropathy and also proposed as potential drug target in order to slow disease progression [26]. Next to TNF itself, soluble TNF receptors 1 and 2 were evaluated in large patient cohort for their prognostic value in predicting the course of the disease [27][28][29]. Induction of TNF via the calcium/calcineurin/NFAT pathway was shown by Canellada and colleagues which was inhibited by tacrolimus [30]. Aomatsu et al. could demonstrate that tacrolimus suppresses TNF-induced CCL2 and CXCL10 expression in human colonic myofibroblasts [31]. CCL2 is next to the soluble TNF receptors another widely discussed and studied prognostic marker in the context of diabetic nephropathy [32][33][34]. A very recent review discussed in more detail the involvement of TNF as well as CCL2 in the context of diabetic nephropathy [35]. Evidence on the beneficial effect of blocking the CCL2/CCR2 signaling cascade is available on the level of animal models as demonstrated by Seok and colleagues in db/db mice [36]. In another animal model on diabetic nephropathy, blockade of the TNF-TNFR2 pathway was shown to have beneficial impact on disease progression [37]. This all supports using tacrolimus in order to slow disease progression in patients with diabetic nephropathy.

The downside of tacrolimus on renal tissue becomes evident when looking at upregulated molecular features in the DN molecular model (Fig 2). Especially molecular features linked to transforming growth factor beta 1 (TGFB1) signaling were upregulated by tacrolimus, namely TGFB1 itself as well as COL4A1, NADPH oxidase 4 (NOX4), or thrombospondin 1 (THBS1). Upregulation of TGFB signaling is one of the major components reported to be responsible for chronic nephrotoxicity of tacrolimus [38]. SMAD family member 2/3 (SMAD2/3)-TGFB signaling mediates renal fibrosis and is next to targeting inflammation in DN a second pillar of current therapeutic strategies in DN [39][40]. Interestingly SMAD7, an intracellular antagonist of TGFB1-mediated fibrosis, was upregulated in our mesangial cell experiment based on tacrolimus treatment [41].

Another pro-fibrotic molecule, namely endothelin 1 (EDN1), was upregulated by tacrolimus in mesangial cells, thus reinforcing the finding that tacrolimus rather activates than inhibits TGF-beta signaling [42].

Based on these findings further studies are warranted stratifying DN patients based on TNF levels in order to identify patients being prone for treatment with low-dose tacrolimus [43][44]. TGFB1 as well as EDN1 should however be used as monitoring markers in order to assess potential nephrotoxic effects of higher doses of tacrolimus.

A further strategy might be to use drugs that block detrimental effects induced by tacrolimus like e.g. blocking TGF-beta signaling cascade with TGFB1 antibodies or using endothelin 1 receptor antagonists which are currently in clinical testing in the context of diabetic nephropathy. Such strategies were already proposed as alternative to conventional strategies of calcineurin inhibitor avoidance, withdrawal, or minimization in the context of renal transplantation [38]. The endothelin receptor antagonist ambrisentan was shown to reduce nephrotoxicity in a rat model of liver transplantation [45]. The angiotensin receptor blocker losartan in addition was shown to significantly decrease TGFB and endothelin levels in human plasma [46][47]. A first trial in human DN patients with tacrolimus was very recently published showing the beneficial effect of tacrolimus on diabetic nephropathy when administered in combination with valsartan [6].

## Conclusions

Inflammatory mechanisms, and in particular the tumor necrosis factor signaling cascade, contributing to progression of diabetic nephropathy might be counterbalanced by tacrolimus treatment. Using TNF as predictive marker and TGFB1 and EDN1 as monitoring markers might be a valuable strategy finding the subset of diabetic nephropathy patients benefitting the most from tacrolimus treatment.
